# Response Mechanisms of Plants Under Saline-Alkali Stress

**DOI:** 10.3389/fpls.2021.667458

**Published:** 2021-06-04

**Authors:** Shumei Fang, Xue Hou, Xilong Liang

**Affiliations:** ^1^Department of Biotechnology, College of Life Science and Biotechnology, Heilongjiang Bayi Agricultural University, Daqing, China; ^2^Department of Environmental Science, College of Agriculture, Heilongjiang Bayi Agricultural University, Daqing, China; ^3^Heilongjiang Plant Growth Regulator Engineering Technology Research Center, Daqing, China

**Keywords:** morphological adaptation, endogenous hormone response, signal transduction, epigenetic regulation, osmotic regulation, saline-alkali stress

## Abstract

As two coexisting abiotic stresses, salt stress and alkali stress have severely restricted the development of global agriculture. Clarifying the plant resistance mechanism and determining how to improve plant tolerance to salt stress and alkali stress have been popular research topics. At present, most related studies have focused mainly on salt stress, and salt-alkali mixed stress studies are relatively scarce. However, in nature, high concentrations of salt and high pH often occur simultaneously, and their synergistic effects can be more harmful to plant growth and development than the effects of either stress alone. Therefore, it is of great practical importance for the sustainable development of agriculture to study plant resistance mechanisms under saline-alkali mixed stress, screen new saline-alkali stress tolerance genes, and explore new plant salt-alkali tolerance strategies. Herein, we summarized how plants actively respond to saline-alkali stress through morphological adaptation, physiological adaptation and molecular regulation.

## Introduction

With the increase in population and the deterioration of natural environments, soil saline-alkalization has become an increasingly serious global problem (Shabala, 2013). According to statistics, approximately 7% of the world’s land (more than 900 million hectares) is threatened by saline-alkalization, and there are no effective measures by which to control its spread (Li et al., 2014). In China, the area of saline-alkali soil has reached 100 million hectares, and the Songnen Plain in northeastern China accounts for 3.73 million hectares and is one of three typical saline-alkali soil distribution areas in the world (the other two are Victoria in Australia and California in the United States) (Feng et al., 2007; Wang et al., 2009). Therefore, soil saline-alkalization is an extensive abiotic stressor and has become a major limiting factor for crop production in global agriculture (Wang H. et al., 2018).

The stress effects of soil saline-alkalization on plants include the effects of both salt stress and alkali stress. According to the salt content and pH value, the degree of salt-alkali conditions is classified as mild (the salt content is less than 3‰, and the pH is 7.1-8.5), moderate (the salt content is 3–6‰, and the pH is 8.5–9.5), or severe (the salt content exceeds 6‰, and the pH value exceeds 9.5) (Oster et al., 1999). Salt stress results mainly from NaCl, Na_2_SO_4_ and other neutral salts. On the one hand, these sodium ions will enter the cell directly through channel and carrier proteins that causes ion toxicity. On the other hand, the high ion concentration outside the cell will reduce the osmotic potential, which drives water molecules out of the cell, leading to physiological drought, that is, osmotic stress. Both of these aspects can cause plant metabolic disorders (Richards, 1947). Alkali stress is induced by NaHCO_3_ and Na_2_CO_3_, which further increase the pH on the basis of salt stress. Therefore, in addition to ionic toxicity and osmotic stress, high pH will severely disturb cell pH stability, destroy cell membrane integrity, and decrease root vitality and photosynthetic function (Zhang et al., 2017; Kaiwen et al., 2020). Many studies have shown that combined salt-alkali stresses result in more serious trophic ion imbalance, reduced osmotic adjustment capacity, inhibition of the antioxidant system, and more serious plant growth inhibition (Amirinejad et al., 2017; Chen et al., 2017; Wang X.-S. et al., 2017; Wang J. et al., 2020). For example, under mild alkali stress, bermudagrass slows metabolic processes such as carbohydrate degradation and N metabolism to maintain basic growth but with a slower growth rate. Moderately and severely alkali-stressed plants will accumulate relatively higher amounts of carbohydrates and significantly increase ROS and MDA contents (Ye et al., 2021). In short, the osmotic stress, oxidative stress, ionic toxicity and high-pH stress caused by mixed salt-alkali stress can destroy the cell membrane structure, inactivate enzyme activity, disrupt the ion balance in plant cells etc. However, most studies have focused mainly on salt stress, and little attention has been given to salt-alkali mixed stress.

Based on the extent and severity of plant damage due to soil salinization and alkalization, studying and summarizing the response mechanisms of plants under salt-alkali stress is very important. The results will contribute to the breeding of resistant varieties and provide an important theoretical basis for the rational utilization of saline-alkali land and the sustainable development of agriculture. Hence, in this work, we review how plants actively respond to salt-alkali stress on the basis of three different aspects: (1) morphological adaptations; (2) physiological adaptations, including osmotic regulation, ion and pH balance, antioxidant effects, and endogenous hormone responses; and (3) molecular regulation, including signal transduction, transcription factor involvement, saline-alkali resistance gene expression, and epigenetic regulation.

## Morphological Adaptations to Salt-Alkali Stress

Under salt-alkali stress, the normal growth, development, and physiological and biochemical metabolism of plants are severely disrupted. When plants are exposed to saline-alkali stress, the roots are the first to perceive the stress information, which is gradually transmitted to the aboveground parts. The root surface area and the root tip number, as well as the leaf area and photosynthetic rate, mainly account for the response of the plant seedling biomass to salt-alkaline stress (An et al., 2021). After long-term exposure to saline-alkali stress, plants can alter their morphology to better adapt to the environment. According to reports, the typical changes in the morphology or anatomy of halophytes in response to salinity mainly include an increase in succulence, leaf pubescence, alterations to the number and size of stomata, a multilayered epidermis, thickening of the cuticle, early lignification, inhibition of differentiation, changes in the xylem vessel diameter and quantity, etc. (Waisel, 1972). Studies on three plant species with different responses to salt stress (*Phaseolus vulgaris*, which is salt-sensitive; *Gossypium hirsutum*, which is moderately salt tolerant; and *Atriplex patula*, which is salt tolerant) have shown that high Na^+^ concentrations significantly reduce the leaf area/plant height ratio. The anatomical structure of the leaves indicated that compared with the other species, the salt-tolerant species *A. patula* had greater leaf thickness due to the increase in the epidermal and mesophyll thickness and increased succulence (Longstreth and Nobel, 1979). Moreover, salt stress led to a decrease in the photosynthetic rate and CO_2_ concentrations in the chloroplasts, as determined by the stomatal and mesophyll conductance (Wang X. et al., 2018). Furthermore, the anatomical structure of *Populus euphratica* under salt stress revealed inhibition of xylem differentiation and developmental changes in normal-sized vessels, which improved the plant tolerance to salinity (Chen and Polle, 2010). In addition, studies on cotton and *Leymus chinensis* have also shown that plants can adapt to salt-alkali stress by increasing their root/shoot ratio and specific root length (Liu B. et al., 2015; Wang Q. H. et al., 2018). Dissections of the root structure showed that stress promoted the maturation of both the hypodermis and endodermis, which formed a well-developed Casparian strip closer to the root apex that is helpful for adaptation (Walker et al., 1984). Moreover, a study on the halophyte plant *Kochia sieversiana* showed that the cotyledon node zone may play a role in salt and alkali tolerance. Xylem sap collected from the above cotyledon node zone contains less Na^+^ and Cl^–^ under both salt and/or alkali stresses. The selective restriction of ion transport conferred by the cotyledon node zone under both salt and alkali stresses may represent a novel mechanism of salt and alkali resistance in halophyte plants (Wang et al., 2019). At the cell level, cell expansion increases along the radial axis in the epidermis and cortex under high salinity, which is controlled by modifying the cell wall structure. Studies have shown that a proper cell wall structure is important for the cell shape, elasticity, cell expansion direction and overall growth in roots, which can be directly regulated by salt stress (Shoji et al., 2006; Duan et al., 2015). However, the latest report showed that in response to stress, root aquaporin activity, rather than changes in the root xylem structure, controlled hydraulic conductance, which provides new mechanistic and functional insights into plant adaptation to stress (Domec et al., 2021). In summary, salt-tolerant plants exhibit increased resistance by altering both aboveground and belowground organs to construct an appropriate morphological structure to adapt to adversity.

## Physiological Adaptation Mechanism of Plants Under Saline-Alkali Stress

### Increasing Osmotic Regulatory Ability via Accumulation of Small-Molecule Organic Compounds

Under saline-alkali stress, sodium ion accumulation in the soil causes the osmotic pressure of the soil to be higher than that of plant cells. Under these environmental conditions, water exits plant cells, which causes osmotic stress and physiological drought. To cope with this adversity, plant cells synthesize and accumulate several small-molecule organic compounds, such as proline, soluble proteins, betaine, sugar, polyols and polyamines, to maintain intracellular water potential (Sun et al., 2019). These substances exert osmoregulatory ability by altering the solvent properties of water, stabilizing the internal osmotic potential, increasing the thermodynamic stability of folding proteins and protecting the macromolecular structure. Sorghum seedlings reportedly adapt to salt-alkali environments by altering the synthesis of small-molecule compounds such as proline and soluble proteins (Sun et al., 2019). In wheat, salt-alkali stress led to increases in proline, soluble sugar and polyol (sorbitol) contents to counteract the adverse salt-alkaline conditions (Lin et al., 2012; Guo et al., 2015). Exogenous application of salicylic acid and nitric oxide has been reported to increase plant salt tolerance by enhancing the synthesis of proline, glycine betaine, and sugars that contribute to the maintenance of the tissue water content in *Vigna angularis* (Ahanger et al., 2019). Taken together, these results show that different plant species and different varieties of the same species can respond to salt-alkali stress through changes in different osmotic adjustment substances.

### Maintaining Na^+^-K^+^ Ion Balance via Channel Proteins and Transporters

**FIGURE 1:**
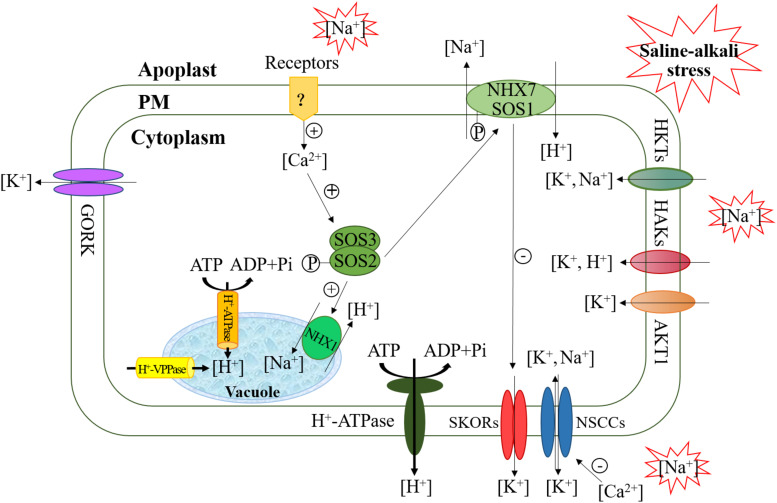
Maintaining Na^+^-K^+^ ion balance via channel proteins and transporters.

The high concentration of sodium ions in the soil under saline-alkali stress can disrupt the dynamic balance of ions in cells, leading to a series of damaging effects on plants, such as destruction of the cell membrane structure, abnormal metabolons in cells and ionic toxicity (Hasegawa, 2013). Plants alleviate the toxicity of sodium ions mainly through excreting sodium ions from cells and sequestering them through ion antiporters such as NHX7 (also named SOS1) within cell membrane and NHX1 within vacuolar membrane, both of whose activity is regulated by calcium-dependent SOS2/SOS3 kinase complexes (Figure 1) (Bahmani et al., 2015). Na^+^ transport is driven by proton-driven forces produced by H^+^-ATPase (located within the cell and vacuolar membranes) and H^+^-VPPase (located within the vacuolar membranes). Saline-alkali stress can increase the activities of H^+^-ATPase and H^+^-VPPase. More H^+^ is pumped into the apoplast and vacuole, which increases the transmembrane electrochemical gradient, and enhances the power of Na^+^ flow from the cytoplasm into the apoplast and vacuole (Deinlein et al., 2014; Ye et al., 2019). Moreover, the SOS2-SOS3 complex is sensitive to Ca^2+^ concentrations, so appropriate Ca^2+^ levels are beneficial to maintaining sodium ion homeostasis under saline-alkali stress.

The potassium (K^+^) concentration is closely related to the regulation of osmosis, the membrane potential, and enzyme activity in plants (Hasanuzzaman et al., 2018). A high cytosolic K^+^/Na^+^ ratio in the cytoplasm is necessary for normal plant growth and development. Under saline-alkali-stress conditions, a large influx of Na^+^ into the cytoplasm can causes the membrane potential to drop below the resting potential, which then activates the K^+^ outflow channel (such as NSCC, GORK, and SKOR) and disrupts the steady-state equilibrium of the K^+^/Na^+^ ratio (Falhof et al., 2016) (Figure 1). There is increasing evidence that some K^+^ channel proteins, including high-affinity K^+^ transporters (HKTs), high-affinity K^+^ absorption transporters channel proteins (HAKs), and Arabidopsis K^+^ transporters (AKT1), are involved in the K^+^ absorption in plants. HKTs are a kind of transport protein specific for Na^+^/K^+^ (type II). Type II HKTs are selective for K^+^ but can also transport Na^+^ under certain conditions (Cotsaftis et al., 2012). Generally, K^+^ absorption is mediated by members of the K^+^ transporter HAK/KUP/KT family (such as HAK5 and KUP7) or members of the shaker family of K^+^ channels (such as AKT1) (Li et al., 2018). A recent study showed that many members of the HAK/KUP/KT family are involved in K^+^ uptake and stress responses in tea plants (Yang T. et al., 2020). Plants overexpressing the *HAK* gene are highly tolerant to salt under low-K^+^ conditions in cytoplasm. However, under the combined conditions of Na^+^ and low K^+^ in plant cytoplasm, HAK expression is inhibited, the activity of the transporter decreases sharply, and plants become very sensitive. The main reason is that Na^+^ depolarizes the plasma membrane (PM), such that its polarity value is higher than that of the K^+^ equilibrium potential, thereby activating the outward-rectifying K^+^ channel (such as SKORs) and leading to K^+^ outflow (Pottosin and Dobrovinskaya, 2014; Bacha et al., 2015). In addition, the activity of PM H^+^-ATPase is another factor that restricts K^+^ absorption. This protein complex is necessary for protons to be actively pumped out of the cell through an ATP-dependent phosphorylation process, generating a proton motif force (pmf) across the PM (Falhof et al., 2016). HAKs then use the pmf produced by H^+^-ATPase for K^+^ absorption because HAKs are usually K^+^/H^+^ symporters (Bose et al., 2013; Pottosin and Dobrovinskaya, 2014). Thus, limiting membrane depolarization (restricting Na^+^ influx or promoting Na^+^ efflux) and enhancing H ^+^-ATPase activity can increase K^+^ absorption via HAKs under salt stress and increase the resistance to low K^+^ under salt stress. Studies in tomato have indicated that inducing *LeHAK5* and supplementing Ca^2+^ during the K^+^ starvation period can counteract the PM depolarization induced by salt stress by inhibiting NSCCs, thereby increasing the absorption of K^+^ (Bacha et al., 2015). From this point of view, maintaining a high K^+^/Na^+^ ratio is an important salt stress adaptation measure. AKT1 participates in high-affinity K^+^ absorption to ensure a constant K^+^ supply, resulting in a high internal-K^+^ to external-K^+^ concentration (Gierth and Mäser, 2007; Demidchik, 2014). When salt stress inhibits the plant’s ability to absorb K^+^ from the soil, increasing the external K^+^ concentration helps alleviate salt stress (Rodrigues et al., 2013). In *Puccinellia tenuiflora*, *PutAKT1* has been characterized as encoding a K^+^ transporter/channel expressed under saline-alkali-stress conditions (Zhao et al., 2016). The overexpression of *PutAKT1* in Arabidopsis seedlings increases K^+^ uptake by cells and reduces Na^+^ accumulation, which proves the role of *PutAKT1* (Ardie et al., 2010). As mentioned above, there are many proteins involved in maintaining high cytoplasmic K^+^/Na^+^ ratios and that are prerequisites for salt stress tolerance because a high cytoplasmic K^+^/Na^+^ ratio can ensure the best cell metabolic function. In addition, some chemicals regulate tolerance to salinity and alkalinity stress by acting on these protein enzymes or channels. γ-aminobutyric acid (GABA) accumulation in Arabidopsis corresponded to increased activity of PM H^+^ ATPase, reduced ROS-induced K^+^ efflux from roots, and lower Na^+^ uptake, which confer salt tolerance to plants (Su et al., 2019). Xu et al. (2019) also reported that GABA can reduce the Na^+^/K^+^ ratio by inducing polyamine generation to enhance salinity-alkalinity stress tolerance in muskmelon. Polyamines were reported to assist in the movement and sequestration of Na^+^ from the cytoplasm to the vacuole and affect K^+^ flux by amending outward- and inward-rectifying K^+^ channels in guard cells and root cells (Pál et al., 2015; Pottosin et al., 2020). Moreover, the application of plant growth-promoting rhizobacteria showed an enhanced capacity to counteract saline-alkaline stress in *Chrysanthemum* plants, which can modify cellular abscisic acid levels, inhibit net K^+^ efflux and concurrently induce net Na^+^ efflux by modulating several Na^+^/H^+^ and K^+^ antiporters/channels (Zhou et al., 2017).

### Maintaining Intracellular pH Stability Through Secreting and Synthesizing Organic Acids

High pH levels in soils occur with increasing saline-alkali stress, which mainly affects the plant root system by destroying root tissue and reducing the root surface area, resulting in root cells losing their normal physiological function (Munns and Tester, 2008; Robin et al., 2016). High-pH stress also causes the mineralization of organic matter such as carbon, nitrogen, phosphorus, and sulfur, which decreases nutrient recycling and availability for plants (Neina, 2019). Studies have shown that many plants can be induced to secrete large amounts of organic acids under saline-alkali stress, which can play a buffering role allowing plants to resist environmental changes and maintain intracellular pH stability and ion balance (Yang et al., 2010; He et al., 2011; Guo-Hui, 2012). Transcriptomic profiling in grapevine roots revealed that the underlying mechanism of the NaHCO_3_-induced synthesis of organic acids may be that phosphoenolpyruvate carboxylase catalyzes the carboxylation of phosphoenolpyruvate with -HCO_3_ to oxaloacetate, which is then converted into oxalate, acetate and malate. The activity of phosphoenolpyruvate carboxylase was regulated by phosphoenolpyruvate carboxylase kinases, which were substantially upregulated by NaHCO_3_ stress (Xiang et al., 2019). A relative study also showed that proton pump H^+^-ATPase may play an important role in organic acid secretion from roots under NaHCO_3_ stress (Guo et al., 2018). A study on tomato indicated that both the roots and leaves of plants maintained the ion balance by enhancing the synthesis of organic acids such as citrate, formate, lactate, acetate, succinate, malate and oxalate under salt and alkali stress. In particular, under alkali stress, large amounts of citrate, malate and succinate were synthesized to compensate for the deficiency of inorganic anions (Wang et al., 2011). These results verified the important role of organic acids in maintaining the cell pH and iron balance. In addition, organic acids are important intermediates of carbon metabolism in plant cells and play other roles in controlling the whole-plant cell physiology, including signaling messengers, modulators of transport across biological membranes, protein modification of acetylation or succinylation and nutrient element uptake from the soil, which can enhance the resistance of plants to a certain extent (López-Bucio et al., 2001; Drincovich et al., 2016; Yang T.Y. et al., 2020).

### Increasing Resistance to Oxidative Damage via Antioxidant Enzymes and Antioxidants

Osmotic stress and ionic stress caused by saline-alkali stress further lead to the generation and accumulation of reactive oxygen species (ROS), such as hydrogen peroxide (H_2_O_2_) and hydroxyl radicals (OH^–^s). Luo et al. (2021) addressed the mechanism of ROS production triggered by salinity. First, NaCl induces the expression of Abscisic Acid-Insensitive 4 (*ABI4*), which can enhance *RbohD* expression but repress *VTC2* expression. Then activated RbohD promotes ROS production while VTC2 repression impairs ROS scavenging. Therefore, this ABI4-RbohD/VTC2 regulatory module positively promotes ROS accumulation (Luo et al., 2021). Accumulated ROS will disrupt the normal physiological functions of cells, resulting in metabolic disorders. There is a set of scavenging systems including antioxidant enzymes and antioxidants for reducing the stress of ROS in plants. The main antioxidant enzymes include superoxide dismutase (SOD), catalase (CAT), peroxidase (POD), glutathione peroxidase (GPX), glutathione reductase (GR) and ascorbic acid peroxidase (APX). SOD is the first line of defense of the antioxidant system in plants and can transform accumulated superoxide molecules into oxygen and H_2_O_2_, after which CAT, APX, and POD convert H_2_O_2_ into water and oxygen. In addition, these enzymes work together to scavenge MDA produced from lipid peroxidation to the protect membrane structure. Antioxidants include mainly glutathione (GSH), ascorbic acid (ASA), mannitol, flavonoids, anthocyanins and vitamin E. These compounds are distributed in different parts of cells to regulate the balance of ROS in cells. Based on the functions of antioxidant enzymes and antioxidants, it is possible to alleviate the injury to cells caused by saline-alkali stress through the cooperation of the two.

A study on rice showed that cell membranes were severely damaged by alkali stress and that the contents of MDA and H_2_O_2_ increased significantly, which stimulated the plants’ antioxidant defense system. The activities of antioxidant enzymes such as SOD, POD, CAT, and APX significantly increased. After a 98% solution of natural anthocyanin exogenous antioxidants was applied, impaired phenotypic characteristics such as wilting, chlorophyll damage and cell death were relieved, and the ROS that accumulated were scavenged (Zhang et al., 2017). A high-salt environment further stimulates the plant response to alkali stress. Sun et al. (2019) reported that under relatively low alkali stress, the activities of SOD and CAT in sorghum seedlings essentially did not change, but under a high-salt environment, with an increase in alkalinity, POD activity increased significantly (Sun et al., 2019). Other studies have also shown that plants can reduce oxidative damage by regulating the activity of antioxidants and antioxidant enzymes to better adapt to salt-alkali stress environments. A study on two different sensitive maize varieties (JY417, a highly salt-tolerant cultivar) and (XY335, a salt-sensitive cultivar) showed that saline-alkali stress could increase both ASA and GSH contents. High ASA and GSH contents ensured the successful cyclization of ASA-GSH, which plays an important role in maintaining protein stability and the structural integrity of the biomembrane system and prevents membrane lipid peroxidation. By cooperating with APX, GR and other antioxidant enzymes, ASA and GSH constitute a cyclical system that effectively removes free radicals, thereby enhancing the antioxidant ability and maintaining the balance of active oxygen metabolism in cells to further effectively alleviate damage caused by salt-alkali stress (Fu et al., 2017). A *Medicago sativa* L. *MsSiR* overexpression enhanced the alkali tolerance of transgenic plants by increasing the GSH content (Sun et al., 2020). Moreover, many studies have shown that the application of exogenous compounds such as, hydrogen sulfide, GABA, 28-homobrassinolide, 24-epibrassinolide, melatonin, salicylic acid, kinetin, jasmonic acid and nitric oxide confers salinity and alkalinity tolerance by upregulating the antioxidant system, ascorbate-glutathione cycle, and glyoxalase system in various plants including soybean, *Malus hupehensis*, muskmelon, *Brassica juncea*, *Pisum sativum*, pepper and tomato cultivars (Ahmad et al., 2017, 2018; Ahanger et al., 2018; Kaur et al., 2018; Alam et al., 2019; Jin et al., 2019; Shams et al., 2019; Kaya et al., 2020; Li H. et al., 2020), which indicates the critical role of the antioxidant system in the stress response.

### Increasing Endogenous Hormone Synthesis by Regulating the Expression of Related Genes

Changes in hormones are an important factor that affect normal plant growth and development under saline-alkali stress. Gibberellin (GA), auxin (IAA), abscisic acid (ABA), cytokinin (CK), ethylene (ET), salicylic acid (SA) and jasmonic acid (JA) are indispensable hormones for plant adaptations to stress, and the concentrations of these hormones are regulated by the expression of hormone-related genes (Ciura and Kruk, 2018; Korver et al., 2018; Kumar et al., 2019). For example, the endogenous GA (GA1 and GA4) content was shown to be less inhibited by saline-alkali stress in a resistant rice variety than in a sensitive variety, and the expression level of GA synthesis-related genes was higher in the former. Several key GA biosynthesis and catabolism-related genes, *OsGA20ox*, *OsGA3ox*, and *OsGA2ox*, in rice were found to participate in the response to saline-alkali stress. The expression of *OsGA20ox1* in the sensitive variety decreased but was maintained at a relatively constant level in the resistant variety. Compared with that in the sensitive variety, the expression of *OsGA20ox3* in the resistant variety was strongly induced in response to saline-alkali stress (Li et al., 2019). Therefore, the resistance mechanisms of plants to salt-alkali stress may be related to the biosynthesis and metabolism of GA. As key hormones for plant growth, IAA and CK accumulate and are widely distributed in root tips to cope with high-pH environments (Xu et al., 2012). The AUX/LAX family of influx carriers and the PIN family of efflux carriers mediate the polar transport of IAA, which is very important for the distribution and accumulation of IAA in plant roots (Blilou et al., 2005; Overvoorde et al., 2010). Under alkaline stress, the expression of the IAA-related genes *ARF5*, *GH3.6*, *SAUR36*, and *SAUR32* and the CK-related gene *IPT5* in the roots of apple rootstocks was significantly induced, and the contents of IAA and CK greatly increased, thereby increasing the alkali resistance of the apple rootstocks (Liu et al., 2019). Another study confirmed that ET can stimulate the expression of AUX1 and IAA biosynthesis-related genes to increase the accumulation of IAA, thereby regulating the inhibition of root elongation affected by alkaline stress (Li et al., 2015). ABA is an important plant hormone for plant growth, development and responses to stress. Studies have shown that in the ABA signaling pathway, the expression of five genes (*SaPYL*_4–1_, *SaPYL*_4–2_, *SaPYL*_4–3_, *SaPYL*_4–4_, and *SaPYL*_5–1_) related to ABA receptors in *Sophora alopecuroides* is downregulated under salt and alkali treatment. These genes regulate stomatal closure by promoting the accumulation of ABA, thereby reducing the inhibitory effect of saline-alkali stress on photosynthesis and allowing plants to better adapt to the stress environment (Guo et al., 2015; Yan et al., 2020). In sorghum plants, the expression levels of *SbNCED3*, *SbPP2C09*, *SbPP2C23*, *SbPP2C52*, *SbPP2C54*, *SbPP2C58*, *SbSAPK1*, *SbSAPK5*, and *SbSAPK9* were significantly upregulated under saline-alkali stress, indicating that these genes may play an important role in ABA signaling under salt-alkali stress (Ma et al., 2019). These results indicate that an increase in hormone content enhances the salt-alkali resistance of plants. The application of exogenous hormones such as ABA, SA, and JA also alleviated harmful effects of salt and alkali stresses on various plants, which further verified the role of hormones in enhancing plant resistance (Ahanger et al., 2019; Ali et al., 2020; Li X. et al., 2020).

## Molecular Mechanisms of Plant Responses to Saline-Alkali Stress

### Activation of Signal Transduction Pathways

The signal transduction pathways of saline-alkali stress mainly include the salt overly sensitive (SOS) pathway, protein kinase pathway and ABA pathway. Among them, the SOS pathway is used for signal transduction under ionic stress, while the protein kinase pathway and ABA pathway are involved mainly in osmotic signal transduction (Zhu, 2016).

The SOS pathway is the first salt-alkali stress signal transduction pathway established in plants and is responsible for the efflux of Na^+^ in cells. In this signaling pathway, the EF-hand chiral calcium-binding proteins Salt Overly Sensitive 3 (SOS3) and SOS3-Like Calcium-Binding Protein (SCaBP8)/Calcineurin B-like Protein 10 (CBL10) are essential for the activation of the SOS signaling pathway. These proteins belong to the CBL/SCaBP protein family, with an EF-hand structural region that can bind calcium ions. Calcium signals can be generated under the stimulation of salt stress (Zhu, 2016). Salt Overly Sensitive 2 (SOS2) is a Ser/Thr protein kinase that acts as an intermediate hub in the SOS signaling pathway. When plants grow in a saline-alkali environment, SOS3 and SCaBP8 activate SOS2 after sensing the calcium signal and combine to form a SOS3-SOS2 protein kinase complex. Activated SOS2 interacts with SOS1 in the PM and then regulates and activates it by phosphorylation, whose Na^+^/H^+^ antitransport activity can export Na^+^ accumulated in the cytoplasm to the outside of the cell. A study has proven that SpSOS1 can improve plant salt tolerance by regulating ion homeostasis and protecting the PM against oxidative damage under salt stress (Zhou et al., 2018). Reverse genetics experiments also verified that SOS1 in PM plays a critical role in the salt tolerance of rice by controlling Na^+^ homeostasis and contributing to the sensing of sodicity stress (El Mahi et al., 2019). In addition, activated SOS2 can also activate the Na^+^/H^+^ antiporter NHX1 (Na^+^/H^+^ Exchanger 1) located within the vacuolar membrane by phosphorylation so that the accumulated Na^+^ in the cytoplasm is sequestered into the vacuole to maintain ion homeostasis. A study has shown that upregulation of *MdNHX1* expression can enhance salt tolerance in apple plants (Zou et al., 2021). Therefore, the SOS signaling pathway is very important for the salt stress response process.

Plants can respond to alkali and salt stress by regulating osmotic stress signaling pathways including mitogen-activated protein kinases (MAPKs), Ca^2+^-dependent protein kinases (CDPKs) and CBL-interacting protein kinases (CIPKs) (Shah et al., 2021; Zhang et al., 2021). The MAPK cascade includes MAPKKK, MAPKK, and MAPKs, which are responsible mainly for transmitting extracellular signals into cells. Upstream MAPKKK is first activated by phosphorylation, and MAPKK and MAPK are in turn phosphorylated and activated sequentially. Activated MAPK can phosphorylate transcription factors and other signaling molecules to regulate the expression of downstream genes (Colcombet and Hirt, 2008; Lee et al., 2015). As Ca^2+^ sensors, CDPKs and CIPKs can directly convert upstream Ca^2+^ signals into downstream phosphorylation signals to initiate further downstream signal transduction. They play an important role in the transcription process and are an important regulatory protein commonly found in plants (Kudla et al., 2018). Typical CDPKs generally have four domains: a variable N-terminal domain (VNTD), Ser/Thr protein kinase domain (PKD), self-inhibitory junction domain (JD) and calmodulin (CaM)-like regulatory domain (CaM-LD). Studies have shown that when plants are stimulated by stresses such as low or high temperatures, high salt and drought, specific Ca^2+^ signals form in the cells. Ca^2+^ directly binds to the CaM-like regulatory domain with an EF-hand-shaped structure at the C-terminus. Changes in the conformation of CDPKs expose the kinase active site and activate kinase activity. In addition, the PKD region activates the substrate by binding ATP or GTP and transferring the γ-phosphate group to the receptor hydroxyl residue, thereby triggering a variety of physiological responses in plants (Kudla et al., 2010; Liese and Romeis, 2013; Yip Delormel and Boudsocq, 2019). According to a report, a tyrosine phosphatase AtPFA-DSP3 can modulate the salt stress response of Arabidopsis by interacting with and dephosphorylating MAPK family members MPK3 and MPK6, suggesting the importance of MAPK moleculars for plant salt tolerance (Xin et al., 2021). *GsMAPK4*-overexpressing soybean plants and *SeMAPKK*-overexpressing Arabidopsis both showed significantly increased tolerance to salt stress, suggesting their positive regulatory effects on the salinity tolerance of plants (Qiu et al., 2019; Rehman et al., 2020). Studies on CDPK members showed that CPK12 was involved in plant adaptation to salt stress by regulating Na^+^ and H_2_O_2_ homeostasis in Arabidopsis (Zhang H. et al., 2018). CPK11 improves salt tolerance in transgenic Arabidopsis plants by regulating Na^+^ and K^+^ homeostasis and stabilizing photosystem II (Borkiewicz et al., 2020). CDPK2 plays a positive role in the salt stress response in potato by promoting ROS scavenging and chlorophyll stability and inducing stress-responsive genes, conferring tolerance to salinity (Grossi et al., 2021). Multiple CIPK members participate in the salt stress response. In two genotypes of switchgrass cultivars (a salt-alkali tolerant genotype and a sensitive genotype), CIPK expression was upregulated mainly in the salt-tolerant cultivars, but the expression in the salt-alkali-sensitive variety was still very low (Zhang et al., 2021). In soybean, GmPKS4 improves soybean tolerance to salt and salt-alkali stresses. The overexpression of *GmPKS4* enhances the scavenging of ROS, osmolyte synthesis, and transcriptional regulation of stress-related genes (Ketehouli et al., 2021). In *Brachypodium distachyon*, BdCIPK31 positively regulates salt stress in stomatal closure, ion homeostasis, ROS scavenging, osmolyte biosynthesis, and transcriptional regulation of stress-related genes (Luo Q. et al., 2017). NtCIPK9 increases transgenic plant salt tolerance by promoting the expression of genes controlling ion homeostasis (Lu et al., 2020). ZmCIPK42 enhances salt tolerance in maize through interaction with calcineurin B-like protein 1 and 4 (ZmCBL1, ZmCBL4), as well as a proteinase inhibitor (ZmMPI) (Chen et al., 2021). PpCIPK1 modulates plant salt tolerance in *Physcomitrella patens* by ionic homeostasis, H_2_O_2_ accumulation, regulating photosynthetic activity. Moreover, the overexpression of *PpCIPK1* could completely rescue the salt-sensitive phenotype of *sos2-1* to wild-type levels in Arabidopsis, suggesting the powerful function of PpCIPK1 (Xiao et al., 2021). Taken together, these results indicate that the MAPK and CDPK cascade signaling pathways may mediate the response of a variety of plants to salt and alkali stress indifferent ways. More extensive research needs to be carried out in the future.

The ABA pathway includes both ABA-dependent and ABA-independent types, which are involved in the regulation of osmosis, ions, and reactive oxygen species under salt stress by regulating the expression of several tolerance genes (Sah et al., 2016; Yu Z. et al., 2020). The ABA-dependent pathway means that gene expression is induced by exogenous or endogenous ABA, and the ABA-independent pathway is a way in which gene expression is not only induced by ABA but also affected by biotic or abiotic stress (Fujii and Zhu, 2009; Cutler et al., 2010; Umezawa et al., 2010). The ABA signaling pathway has four core components: (1) a pyrabactin resistance 1/PYR1-like/ABA receptor regulatory component (PYR1/PYL/RCAR), which is the main receptor of ABA; (2) 2C-type protein phosphatases (PP2Cs), which is negative regulators of ABAs; (3) sucrose non-degradable related protein kinase 2 (SnRK2s), which is a unique Ser/Thr protein kinase; and (4) an ABA-response element (ABRE)-binding protein (AREB)/ABRE-binding factor (ABF) (Kulik et al., 2011; Nakashima and Yamaguchi-Shinozaki, 2013; Tian et al., 2015; Singh et al., 2016). In the absence of ABA or under low ABA concentrations, PP2C can interact with dephosphorylated SnRK2 and inhibit SnRK2 activity, thereby inhibiting ABA signaling. When ABA accumulates in plants under adverse stress conditions, the receptor RCAR binds to ABA and competitively binds to PP2Cs to release SnRK2. Activated SnRK2s then activate and phosphorylate the downstream transcription factor AREB/ABF and initiate the ABA response to regulate various processes of plant growth and development under adverse stress conditions (Fujita et al., 2013; Nakashima and Yamaguchi-Shinozaki, 2013). Studies have shown that, among the 10 members of SnRK2 family in Arabidopsis, the expression of SnRK2.1-SnRK2.10 except SnRK2.9 can be induced by NaCl (Kobayashi et al., 2004). Overexpression of *PtSnRK2.5* and *PtSnRK2.7* in Arabidopsis increased the tolerance of the transgenic plants to salt stress (Song et al., 2016). Moreover, overexpression of a novel gene, *AsSnRK2D*, in tobacco significantly improved the plant tolerance to dehydration or salinity stress. The molecular mechanism might be attributed to the significantly upregulated transcripts of several environmental stress-inducible genes, including dehydrins, cell signaling components, transcription factors, antioxidative enzymes, and proline biosynthesis (Xiang et al., 2020). In addition, GsSKP21, as a *Glycine soja* S-phase kinase-associated protein, plays a critical regulatory role in the ABA-mediated stress response. Overexpression of *GsSKP21* in Arabidopsis dramatically increased the plant tolerance to alkali stress and mediated ABA signaling by altering the expression levels of the ABA signaling-related and ABA-induced genes (Liu A. et al., 2015).

### Induction of Transcription Factor Expression

During signal transduction in response to salt-alkali stress, transcription factors serve as a bridge between stimulus signals and associated genes. They receive upstream signals and regulate the expression of related downstream resistance genes by binding to their corresponding *cis*-regulatory sequences. Transcriptome analysis of switchgrass and alfalfa indicated that the expression levels of many transcription factors were significantly modified in response to saline-alkaline stress. They belong to major transcription factor families such as AP2/ERF, NAC, HD-zip/bZIP, MYC/MYB, WRKY, and bHLH, many of whose members have been shown to be related to the salt-alkali stress response (An et al., 2016; Wang J. et al., 2020; Shah et al., 2021; Zhang et al., 2021).

The AP_2_/ERF transcription factor family is a large family unique to plants with at least one or two highly conserved DNA-binding domains, which are involved in regulating plant growth, development and responses to abiotic stress (Phukan et al., 2017). GsERF6 significantly enhanced plant tolerance to alkaline stress in transgenic Arabidopsis, probably by inducing plant hormones such as ABA and ET as signaling molecules to activate a number of hormone- and stress-responsive genes, such as *RAB18*, *RD29A*, *RD29B*, and *COR47* genes, and some ERF-like genes (Yu et al., 2016). ItERF can improve *Arabidopsis thaliana* salt tolerance by activating the expression of stress-related genes through binding to the GCC-box (Wu J. et al., 2019). Genome-wide analysis in adzuki bean showed that the expression of 13 ERF genes was induced in response to saline-alkaline stress. Overexpression of *VaERF3* in transgenic Arabidopsis enhances saline-alkaline tolerance by activating the transcription of stress-responsive genes in an ABA-dependent manner (Li W. Y. et al., 2020). GsERF71 enhances the tolerance of transgenic Arabidopsis plants to alkaline stress by upregulating the expression levels of H^+^-ATPase and by modifying auxin accumulation in transgenic plants (Yu et al., 2017). In rice, OsSTAP1 functions as an AP2/ERF transcriptional activator, and plays a positive role in salt tolerance by decreasing the Na^+^/K^+^ ratio and maintaining cellular redox homeostasis (Wang Y. et al., 2020). Other ERF family members such as LkERF-B2, ERF38, MbERF11 are also reported to improve salinity tolerance (Cao et al., 2019; Cheng et al., 2019; Han et al., 2020).

Recent studies have shown that bZIPs participate in bZIP transcriptional activation under bicarbonate-alkali stress and alter stress-related physiological indicators (such as reducing the accumulation of MDA and increasing both the activity of POD and the content of chlorophyll) and gene expression (excess GsbZIP67 expression in alfalfa) to improve salt-alkali tolerance (Wu et al., 2018). Members of the bZIP transcription factor family have been identified in a variety of higher plants. Plant-specific HD-Zip I transcription factor MdHB-7 regulates salt tolerance in transgenic apple (*Malus domestica*). The overexpression of MdHB-7 reduced the salt stress-induced damage, maintained ion homeostasis, and promoted the detoxification of ROS, while *MdHB-7* RNAi lines showed the opposite performance (Zhao et al., 2021). *Populus nigra* PnHB7 transcription factor overexpression in tobacco also improved the resistance of transgenic plants to salt stress. Transcriptome analysis of overexpressed tobacco showed that hormone-related protein genes, oxidase genes and transcription factor protein genes in the ABA signaling pathway were significantly upregulated, suggesting that PnHB7 plays an important role in the ABA regulation pathway (Yu X. et al., 2020). A novel bZIP transcription factor, *ChbZIP1*, from the alkaliphilic microalgae *Chlorella* sp. BLD has been reported to increase the alkali resistance of plants. Overexpression of *ChbZIP1* in Arabidopsis showed that ChbZIP1 can enhance plant adaptation to alkali stress through the active oxygen detoxification pathway, suggesting its promising potential in genetically improving plant tolerance to alkali stress (Qu et al., 2021). bZIP transcription factors are similar to MYB/MYC transcription factors in terms of their regulation, participation in ABA-dependent pathway signal transduction, and perception of stress signals to regulate gene expression.

Under long-term salt-alkali stress, the members of the MYB transcription factor family exhibited the most significant changes in alfalfa, and the expression of most MYB transcription factors tended to increase (Coskun et al., 2016). In the analysis of alfalfa transcripts, the MYB family was the transcription factor family whose members presented the second strongest response to salt stress after AP_2_ members, indicating that MYBs play an important role in alfalfa salt-alkali resistance (Postnikova et al., 2013). Transcriptional profiling reveals that the MYB transcription factor MsMYB4 contributes to the salinity stress response of alfalfa. The introduction of *MsMYB4* significantly increased salinity tolerance in transgenic Arabidopsis plants in an ABA-dependent manner (Dong et al., 2018). *GmMYB68* overexpression enhanced salt-alkali resistance in soybean, whose osmotic adjustment and photosynthetic rates were stronger than those of GmMYB68-RNAi and wild-type plants. Importantly, the overexpression of *GmMYB68* also increased the grain number and 100-grain weights under salt stress, indicating the value of its practical application to increase crop yields (He et al., 2020). GmMYB3a, as another MYB transcription factor, showed a negative regulatory effect on soybean response to salt-alkali stress. Overexpression of *GmMYB3a* reduced physiological parameters, including soluble sugar, free proline, and chlorophyll contents, and photosynthetic rate and downregulated a set of key genes associated with plant defense signal pathways in the transgenic plants (He et al., 2018). *TaMYB86B* encodes an R2R3-type MYB transcription factor. Overexpression of *TaMYB86B* can increase the salt resistance of wheat by regulating ion homeostasis to maintain an appropriate osmotic balance and decrease ROS levels (Song et al., 2020b). The R2R3-MYB transcription factor AtMYB49 modulates salt tolerance in Arabidopsis by modulating the cuticle formation and antioxidant defense. Overexpression of *AtMYB49* in Arabidopsis increases Ca^2+^ accumulation in leaves, reduces oxidative damage and improves the membrane integrity through upregulation of the expression of genes encoding PODs and SODs and LEAs and decreases non-stomatal leaf water loss by positively modulating cutin deposition in leaves through upregulation of genes classified into cutin, suberin and wax biosynthesis during salt stress. These actions are probably achieved through ABA-dependent signaling pathways with the involvement of at least ABF3 and ABI5 (Zhang P. et al., 2020).

The WRKY gene family, as a plant-specific transcription factor group, plays important roles in many different response pathways to saline and alkali stresses (Li W. et al., 2020). A large number of WRKYs have been functionally characterized in plants. In sweet potato (*Ipomoea batatas* L.), 79 IbWRKY transcription factors were identified and 35 *IbWRKY* genes showed significantly expression changes upon NaCl treatment (Qin et al., 2020). In the sugar beet genome, a total of 58 putative *BvWRKY* genes were identified. *BvWRKY10* in shoots and *BvWRKY16* in roots were remarkably upregulated by alkaline stress (Wu G. Q. et al., 2019). In *Iris lactea* var. *chinensis*, the expression of *IlWRKY1* was notably increased under NaCl stress, suggesting that IlWRKY1 may be involved in *I. lactea* var. *chinensis* sodium salt responses (Tang et al., 2018). Overexpression of *SlWRKY28* improved the tolerance of *Populus davidiana* × *Populus bolleana* to saltine-alkaline stress by inducing regulation of the enzyme gene in the ROS scavenging pathway (Wang X. et al., 2020). *GmWRKY16* could be induced to express by salt in soybean. GmWRKY16 transgenic Arabidopsis showed improved salt tolerance by activating the expression of *AtWRKY8*, *KIN1*, and *RD29A* in the ABA pathway (Ma et al., 2018). In alfalfa, *MsWRKY11* was upregulated in response to salinity and alkalinity stresses. Overexpression of the *MsWRKY11* gene enhanced salt tolerance in soybean by increasing soluble protein and proline contents and reducing ROS levels, but the detailed regulatory mechanisms remain to be further investigated (Wang Y. et al., 2018). *MdWRKY100* overexpression enhanced salt tolerance in *M. domestica*, which was upregulated by the miR156/SPL regulatory module (Ma et al., 2021). *MxWRKY64*, which is a new WRKY transcription factor gene from *Malus xiaojinensis*, was induced to express by salt stress in *M. xiaojinensis* seedlings. Overexpression of *MxWRKY64* in transgenic *A. thaliana* contributed to morphological and physiological indicators, suggesting its important role in the response to salt stress (Han et al., 2021). However, not all WRKYs found will improve salt or alkali tolerance. The transcription factor SbWRKY50 from sweet sorghum is negatively involved in the salt response, reducing salt tolerance in *A. thaliana* by directly binding to the upstream promoter of SOS1 and HKT1 to control ion homeostasis (Song et al., 2020a). The maize *ZmWRKY114* gene also negatively regulates salt-stress tolerance in transgenic rice by attenuating ABA signaling (Bo et al., 2020).

The plant-specific NAC transcription factor family has received much attention in responses to salinity and alkali stress (Marques et al., 2017; Khan et al., 2018). Plant adaptation to environments with high salinity and alkalinity may be related to the different patterns of action of NAC factors. ThNAC13 was reported to improve salt and osmotic stress tolerance in Transgenic *Tamarix* and Arabidopsis by enhancing the ROS-scavenging capability and adjusting the osmotic potential (Wang L. et al., 2017). Overexpression of *GsNAC019* in Arabidopsis resulted in enhanced tolerance to alkaline stress at the seedling and mature stages, but reduced ABA sensitivity, implying that GsNAC019 may contribute to alkaline stress tolerance via the ABA signal transduction pathway and regulate the expression of downstream stress-related genes such as *AtRD29B* (Cao et al., 2017). Under salt stress, the *MdNAC047* gene was significantly induced and MdNAC047 directly activated the expression of *MdERF3* by binding to its promoter, facilitating ethylene release, which enhanced the plant tolerance to salt stress (An et al., 2018). Zhang et al. (2015) revealed that wheat TaNAC47 enhanced salt tolerance by interacting with ABRE *cis*-elements, implying that TaNAC47 may participate in the ABA-dependent signaling pathway. Moreover, *PeNAC036* overexpression enhanced Arabidopsis plant salt stress responses, while transgenic plants overexpressing *PeNAC034* in Arabidopsis and *PeNAC045* in poplar were sensitive to salt (Lu et al., 2018). These results indicate versatile roles of NAC in the responses to salt and alkali stress in plants.

### Upregulation of Salt-Alkali Resistance Gene Expression

Salt-alkali stress induces the expression of related resistance genes, which are involved mainly in osmotic regulation, ion homeostasis, oxidative activity and hormone signal transduction. Studies have shown that in response to high-pH stress, the expression of genes involved in ionic homeostasis and starch and sucrose metabolism is significantly upregulated in cotton. These genes in turn induce plant hormone signal transduction and key enzyme activity to counteract ion toxicity (Zhang B. et al., 2018).

Resistance genes related to osmotic regulation are involved mainly in the synthesis of key enzymes needed for osmotic regulation. Studies have shown that *MsGSTU8* in transgenic tobacco increases the soluble sugar content under salt-alkali stress. In addition, the expression of genes related to proline biosynthesis, including *NtP5Cs*, *NtLEA5*, and *NtLEA14*, was upregulated. This shows that the expression of genes involved in the synthesis of osmotic substances increases plant resistance (Du et al., 2019). Δ^1^-pyrroline-5-carboxylate synthetase (P5CS) is a key enzyme involved in the biosynthesis of proline. Upregulated expression of *PutP5Cs* unigenes under salt-alkali stress significantly increased the content of proline in *P. tenuiflora*, mediating osmotic adaptation to saline-alkaline soil (Ye et al., 2019).

Some saline-alkali resistance genes encoding reverse transport protein/channel ions, including the *PutAKT1*, *PutCAX1*, *PutNHA1*, *HKT*, and *NHX* genes, play an important role in the response to ion stress. Studies have shown that *PutAKT1* is involved in mediating K^+^ absorption. The expression of *PutAKT1* in Arabidopsis increases the K^+^ content and decreases the Na^+^ content in the shoots and roots (Ardie et al., 2010). Under saline-alkali stress, the expression of *NHX2* is upregulated in cotton root and leaf tissues (Zhang et al., 2016). *OsHKT1;4* and *OsHKT1;5* in rice can alleviate the effects of excessive Na^+^ and reduce ion toxicity (Kobayashi et al., 2017). In addition, salt stress induces the expression of *AtHKT1;1* in Arabidopsis, reduces the Na^+^ content in plants and reduces toxicity (An et al., 2017).

Antioxidant-related genes in plants induce the synthesis of key antioxidant enzymes such as SOD, POD, CAT, and GSH, thereby removing active oxygen to protect organisms from oxidative damage. Glutathione *S*-transferase (GST) is a large multifunctional protective cellular enzyme in plants. Members of the GST family quench reactive molecules and catalyze the binding of GSH to hydrophobic and electrophilic substrates, thereby protecting cells from oxidative damage (Liu et al., 2017; Kayum et al., 2018). Overexpression of the *MsGSTU8* gene in transgenic tobacco induced the expression of three ROS detoxification-related genes (*NtSOD*, *NtPOD*, and *NtCAT*), which in turn reduced the accumulation of ROS and the content of MDA; increased the activity of SOD, POD, and CAT; and improved the resistance of transgenic tobacco to salt-alkali stress (Du et al., 2019). Based on a large number of studies on the tolerance of plants under salt/alkali stress, a type of plant metallothionein (MT) related to the resistance of plants under extreme environmental stress has been identified. MTs compose a family of low-molecular weight (7–10 kDa) proteins that are rich in Cys and can bind to metals in a variety of organisms. When plants are exposed to metal and/or saline-alkali stress, MT function is triggered in the plants. MTs in plants can be divided into four types according to the distribution of Cys: MT1, MT2, MT3, and MT4 (Cobbett and Goldsbrough, 2002). The cysteine within MTs directly participates in the process of removing ROS, which reduces the accumulation of ROS in cells (Nzengue et al., 2012). For example, the *SsMT2* gene can improve a plant’s H_2_O_2_-scavenging ability and can maintain H_2_O_2_ at low levels in transgenic Arabidopsis, thereby improving tolerance (Jin et al., 2017). This shows that MT2 may have an antioxidant effect by participating in reducing the accumulation of ROS, thereby reducing cell damage, and that MT2 plays no part in metal sequestration. Phosphoenolpyruvate carboxylase (PEPC) is a strictly regulated cytoplasmic enzyme that plays a role in carbon fixation during photosynthesis. The role of PEPC kinase (PPCK) is to control the phosphorylation state and biological activity of PEPC. Studies have shown that PEPC/PPCK plays an important role in responses to environmental stress. One of the best examples is the significant increase in PPCK activity under salt stress (García-Mauriño et al., 2003; Peng et al., 2012; Monreal et al., 2013). Studies of alfalfa plants expressing the *GsPPCK3* gene have shown that under alkaline stress, transgenic alfalfa plants present increased resistance (Sun et al., 2014). In addition, transglutaminases (TGases), which are enzymes catalyzing the posttranslational modification of proteins, were induced by salt stress in cucumber. Ectopic overexpression of *CsTGase* in tobacco showed that *CsTGase* enhanced salt tolerance by regulating antioxidant activities, the Na^+^/K^+^ balance, and PA metabolism in transgenic lines (Zhong et al., 2020). Some genes associated with salinity and alkalinity adversity response in plants are seen in Table 1.

**TABLE 1:** List of genes associated with salinity and alkalinity adversity response in plants.

Gene name	Source of species	Regulatory functions	Types of saline-alkali stress	Tolerance	Transgenic species	References
*VaERF3*	Adzuki bean	Promote proline accumulation; decrease MDA and ROS contents; promote the expression of stress responsive genes	NaHCO_3_	+	Overexpression in Arabidopsis	Li W. Y. et al., 2020
*MsGSTU8*	Alfalfa (*Medicago sativa*)	Maintain the chlorophyll content; improve antioxidant enzyme activity and soluble sugar levels; reduce ion leakage, ROS accumulation and MDA content	Na_2_CO_3_ + NaHCO_3_	+	Overexpression in tobacco	Du et al., 2019
*MsMYB4*	Alfalfa (*Medicago sativa*)	Increase the plants’ salinity tolerance in an ABA-dependent manner	NaCl	+	Overexpression in Arabidopsis	Dong et al., 2018
*MsWRKY11*	Alfalfa	Increase the contents of chlorophyll, proline, soluble sugar, SOD and CAT; reduce the relative electrical conductivity, the contents of MDA and ROS; Increase pods per plant, seeds per plant and 100-seed weight	NaCl or Na_2_CO_3_ + NaHCO_3_	+	Overexpression in soybean	Wang X. et al., 2018
*MsSiR*	Alfalfa (*Medicago sativa*)	Increase the GSH content	NaHCO_3_	+	Overexpression in alfalfa	Sun et al., 2020
*MdHB-7*	Apple (*Malus domestica*)	Reduce root damage; maintained ion homeostasis; detoxify ROS	NaCl	+	Overexpression and RNAi in apple	Zhao et al., 2021
*MdWRKY100*	Apple (*Malus domestica*)	Increase chlorophyll content, reduce H_2_O_2_ and MDA levels	NaCl	+	Overexpression in Apple	Ma et al., 2021
*MdNAC047*	Apple (*Malus hupehensis*)	Directly activate the expression of *MdERF3* and facilitate ethylene release	NaCl	+	Overexpression in apple and Arabidopsis	An et al., 2018
*AtHKT1*	Arabidopsis	Reduce Na^+^ toxicity	NaCl	+	Gene knockout and complementation in Arabidopsis	An et al., 2017
*CPK12*	*Arabidopsis*	Regulate Na^+^ and H_2_O_2_ homeostasis	NaCl	+	RNAi mutation in Arabidopsis	Zhang H. et al., 2018
*AtMYB49*	Arabidopsis	Modulate the cuticle formation and antioxidant defense	NaCl	+	Gene knockout and overexpression in Arabidopsis	Zhang P. et al., 2020
*BdCIPK31*	*Brachypodium distachyon*	Promote stomatal closure, ion homeostasis, ROS scavenging, osmolyte biosynthesis, and regulation of stress-related genes	NaCl	+	Overexpression in tobacco	Luo Q. et al., 2017
*ChbZIP1*	*Chlorella* sp. BLD	Active stress response gene such as *GPX1*, *DOX1*, *CAT2*, and *EMB* by binding G-box 2 motif and active oxygen detoxification pathway	NaHCO_3_	+	Overexpression in Arabidopsis	Qu et al., 2021
*CsTGase*	Cucumber	Regulate PA metabolism and Na^+^/K^+^ balance	NaCl	+	Overexpression in tobacco	Zhong et al., 2020
*GsPPCK3*	*Glycine soja*	Decrease ion leakage and MDA content, increase chlorophyll content and root activity	NaHCO_3_	+	Overexpression in *Medicago sativa*	Sun et al., 2014
*ItERF*	*Iris typhifolia*	Activate the expression of stress-related genes through binding to the GCC-box	NaCl	+	Overexpression in Arabidopsis	Wu J. et al., 2019
*LkERF-B2*	*Larix kaempferi*	Higher content of chloroplast pigments; the activity of SOD and POD was also enhanced	NaCl	+	Overexpression in Arabidopsis	Cao et al., 2019
*ZmCPK11*	Maize (*Zea mays*)	Regulate Na^+^ and K^+^ homeostasis and stabilizing photosystem II	NaCl	+	Overexpression in Arabidopsis	Borkiewicz et al., 2020
*ZmCIPK42*	Maize (*Zea mays*)	Interaction with ZmCBL1, ZmCBL4, and ZmMPI	NaCl	+	Overexpression in maize and Arabidopsis	Chen et al., 2021
*ZmWRKY114*	Maize (*Zea mays*)	Regulate stress- and ABA-related gene expression	NaCl	−	Overexpression in rice	Bo et al., 2020
*MxWRKY64*	*Malus xiaojinensis*	Higher activities of SOD, POD, and CAT, higher contents of proline and chlorophyll, while MDA content was lower	NaCl	+	Overexpression in Arabidopsis	Han et al., 2021
*NtCIPK9*	*Nitraria tangutorum*	Promote the expression of genes controlling ion homeostasis	NaCl	+	Overexpression in Arabidopsis	Lu et al., 2020
*AsSnRK2D*	Oat	Modulate the expression of stress-inducible genes including dehydrins, cell signaling components, transcription factors, antioxidative enzymes, and proline biosynthesis	NaCl	+	Overexpression in tobacco	Xiang et al., 2020
*PpCIPK1*	*Physcomitrella patens*	Regulate ionic homeostasis, H_2_O_2_ accumulation, photosynthetic activity	NaCl	+	Gene knockout in *P. patens*	Xiao et al., 2021
*PtSnRK2.5 PtSnRK2.7*	Poplar (*Populus trichocarpa*)	Increase survival rates and metabolic regulatory genes expression	NaCl	+	Overexpression in Arabidopsis	Song et al., 2016
*PeNAC036*	*Populus euphratica*	Upregulate the expression of *COR47*, *RD29B*, *ERD11*, *RD22* and *DREB2A*	NaCl	+	Overexpression in Arabidopsis	Lu et al., 2018
*PeNAC034*	*Populus euphratica*	Downregulate the expression of *COR47*, *RD29B*, *ERD11*, *RD22* and *DREB2A*	NaCl	−	Overexpression in Arabidopsis	Lu et al., 2018
*PeNAC045*	*Populus euphratica*	Decrease net photosynthesis rate, stomatal conductance and transpiration rate	NaCl	−	Overexpression in poplar	Lu et al., 2018
*PnHB7*	*Populus nigra*	Increase expression of some TFs and stress-defense-related genes in ABA pathway	NaCl	+	Overexpression in tobacco	Yu X. et al., 2020
*CDPK2*	Potato	Promote ROS scavenging, chlorophyll stability and salt-tolerant gene induction	NaCl	+	Overexpression in potato	Grossi et al., 2021
*OsHKT1;5*	Rice	Mediate Na^+^ exclusion in the phloem to prevent Na^+^ transfer to young leaf blades	NaCl	+	T-DNA insertion mutation in rice	Kobayashi et al., 2017
*OsSTAP1*	Rice	Decrease the Na^+^/K^+^ ratio, increasing the activities of antioxidant enzymes	NaCl	+	Overexpression in rice	Wang Y. et al., 2020
*SeMAPKK*	*Salicornia europaea*	Improve plant growth	NaCl	+	Overexpression in Arabidopsis	Rehman et al., 2020
*SlWRKY28*	*Salix linearistipularis*	Regulating enzyme genes associated with ROS scavenging pathway	NaHCO_3_	+	Overexpression in *Populus davidiana* × *P. bolleana*	Wang X. et al., 2020
*SpSOS1*	*Sesuvium portulacastrum*	Regulate ion homeostasis and protecting the plasma membrane against oxidative damage	NaCl	+	Overexpression in Arabidopsis	Zhou et al., 2018
*GsMAPK4*	Soybean (*Glycine soja*)	Improve plant growth	NaCl	+	Overexpression in soybean	Qiu et al., 2019
*GmWRKY16*	Soybean (*Glycine max*)	Regulate transcription of the stress- and ABA-responsive genes with ABA and proline accumulation, and MDA decrease.	NaCl	+	Overexpression in Arabidopsis	Ma et al., 2018
*GmPKS4*	Soybean (*Glycine max*)	Enhance the scavenging of ROS, osmolyte synthesis, and the transcriptional regulation of stress-related genes	NaCl + NaHCO_3_	+	Overexpression in soybean and Arabidopsis	Ketehouli et al., 2021
*GmMYB68*	Soybean (*Glycine max*)	Adjust osmotic and photosynthetic processes; increased grain number and 100-grain weight	NaCl or Na_2_CO_3_	+	Overexpression and RNAi in soybean	He et al., 2020
*GmMYB3a*	Soybean (*Glycine max*)	Free Pro content decrease; photosynthesis rate decrease; reduced the transcription of stress related genes	NaCl or Na_2_CO_3_	−	Overexpression in soybean	He et al., 2018
*GsSKP21*	Soybean (*Glycine soja*)	Altering the expression of ABA signaling-related and ABA-induced genes	NaHCO_3_	+	Overexpression in Arabidopsis	Liu A. et al., 2015
*GsERF6*	Soybean (*Glycine soja*)	ABA and ET signaling pathways	NaHCO_3_	+	Overexpression in Arabidopsis	Yu et al., 2016
*GsERF71*	Soybean (*Glycine soja*)	Upregulate H^+^-ATPase expression and by modifying auxin accumulation	NaHCO_3_	+	Overexpression in Arabidopsis	Yu et al., 2017
*GsNAC019*	Soybean (*Glycine soja*)	Regulate expression of stress-responsive genes, decreases plant ABA sensitivity, recognize *AtRD29B* promoter	NaHCO_3_	+	Overexpression in Arabidopsis	Cao et al., 2017
*SsMT2*	*Suaeda salsa*	Directly bind ion and trigger other genes’ function, or indirectly improve ROS scavenging	NaCl or NaHCO_3_	+	Overexpression in Arabidopsis	Jin et al., 2017
SbWRKY50	Sweet sorghum	Directly binding to the upstream promoter of *SOS1* and/or *HKT1* to control ion homeostasis	NaCl	−	Overexpression in Arabidopsis and sweet sorghum	Song et al., 2020a
*ThNAC13*	*Tamarix hispida*	Enhance the ROS-scavenging capability and adjusting osmotic potential	NaCl	+	Overexpression and RNAi in *T. hispida*; Overexpression in Arabidopsis	Wang L. et al., 2017
*TaMYB86B*	Wheat	Regulate ion homeostasis, maintain osmotic balance and decrease ROS levels	NaCl	+	Overexpression in wheat	Song et al., 2020b
*TaNAC47*	Wheat	Up-regulate stress responsive genes in ABA pathway, including *AtRD29A*, *AtRD29B*, *AtCOR47*, *AtRD20*, *AtGSTF6*, and *AtP5CS1* by binding ABRE *cis*-element	NaCl	+	Overexpression in Arabidopsis	Zhang et al., 2015

### Epigenetic Changes

Plant tolerance to saline-alkali stress also involves the regulation of epigenetic mechanisms, mainly DNA methylation and histone modification. These heritable changes can influence chromatin structure, which results in gene expression alterations without changes in the underlying DNA sequence (Verhoeven et al., 2010; Zhang et al., 2010; Ganguly et al., 2017; Hewezi, 2018).

In plants, DNA methylation commonly occurs at cytosine sites within CpG, CpHpG and CpHpH sequence contexts (He et al., 2011; Lang et al., 2015). Cytosine methylation is established through *de novo* methylation and maintenance methylation mediated by RNA-directed DNA methylation (RdDM) pathway and several DNA methyltransferases such as DRM1, DRM2, MET1 and CMT3 (Matzke and Mosher, 2014). Methyl groups on these cytosines can also be removed by either passive DNA demethylation (failure to maintain methylation after replication) or active DNA demethylation mediated by members of the bifunctional DNA glycosidase subfamily including Demeter (DME), Repressor of Silencing 1 (ROS1) and Demeter-like (DML) (Gehring et al., 2006; Morales-Ruiz et al., 2006; Zhu, 2009; Lang et al., 2017; Park et al., 2017). By methylation/demethylation processes, DNA methylation in plants can be dynamically regulated and maintained at a proper level. Previous studies have shown that gene expression in plants can be altered through DNA hypomethylation or hypermethylation to adapt to salt-alkali-stress environments (Marconi et al., 2013; Viggiano and de Pinto, 2017). In a salt-tolerant *Setaria italica* L. cultivar, the expression of stress-responsive genes is correlated with DNA demethylation events under salinity stress (Pandey et al., 2017). *NtGPDL* gene demethylation within the coding sequence of tobacco was shown to be induced by salt stress, which increased *NtGDPL* gene expression (Choi and Sano, 2007). However, in *Medicago truncatula* and olive plants, salinity stress increased DNA methylation levels, which regulated the expression of several stress-responsive genes as a stress-adaptive response (Yaish et al., 2018; Mousavi et al., 2019).

Nucleosome core complex histones undergo various posttranslational modifications, including acetylation, phosphorylation, ubiquitination, biotinylation, and sumoylation, which influence chromatin structure and thus determine the expression levels of some genes (Nathan et al., 2006; Camporeale et al., 2007; Sridhar et al., 2007; Luo M. et al., 2017; Su et al., 2017). Therefore, it is understandable that stress-induced gene regulation is associated with histone modifications. Under salt stress, changes in histone modification are involved in the regulation of plant growth and development. Salt stress was shown to increase flowering inhibitor Flowering Locus C (FLC) expression in Arabidopsis by reducing the interaction of the floral initiator Shk1 kinase binding protein 1 (SKB1) with chromatin and by reducing H4R3 symmetric dimethylation (H4R3sme2) levels, thereby regulating flowering time (Zhang et al., 2011). In maize roots, salt stress induces changes in histone acetylation within the promoter regions of cell cycle-related genes (Zhao et al., 2014; Zhou et al., 2014). Elevated acetylation levels at H3K9 and H3K27 sites lead to transcriptional activation of POX-encoding genes in *Beta vulgaris* and *Beta maritima* under salt-stress conditions (Yolcu et al., 2016). The transcription factor MsMYB4 is an important component of the response of alfalfa to salinity stress. The activation of MsMYB4 was reported to be associated with a reduction in the DNA methylation status and an increase in histone H3K4 trimethylation and H3K9 acetylation in the promoter (Dong et al., 2020).

Many salt-responsive small RNAs have been documented in plants, including miRNAs and siRNAs. Their functions in response to salt stress in plants were reviewed by Kumar et al. (2018). Further small RNA and degradome sequencing of superior stress-tolerant wheat revealed 219 novel and 98 known miRNA sequences. A number of target genes of the miRNAs participating in multiple processes have been identified, among which jasmonate signaling and carbohydrate metabolism are important for salinity tolerance, and proton transport is vital for alkalinity tolerance (Han et al., 2018). In a typical halophyte, *Reaumuria soongorica*, 13 novel miRNAs were discovered under salt stress. miRNA-mRNA integrated analysis revealed that miRNAs regulate the network response to salt stress during seed germination through GA, auxin, and ABA signaling pathways (Zhang H. et al., 2020). In addition, a comparative transcriptome analysis revealed some new lncRNAs in sweet sorghum, including lncRNA13472, lncRNA11310, lncRNA2846, lncRNA26929, and lncRNA14798. They potentially participate in the response to salt stress by regulating the expression of target genes related to ion transport, protein modification, transcriptional regulation, and material synthesis and transport (Sun et al., 2020). These reports indicated the importance of epigenetic modification in the response to saline and alkali stress.

Further work is needed to expound upon the epigenetic regulatory mechanisms of plants in response to stress, especially saline-alkali stress. Additional enzymes or proteins need to be further explored, addressing how these enzymes, small RNAs and their interacting proteins work together to control DNA methylation and histone modification at specific loci that regulate stress-responsive gene expression.

### Perspectives

**FIGURE 2:**
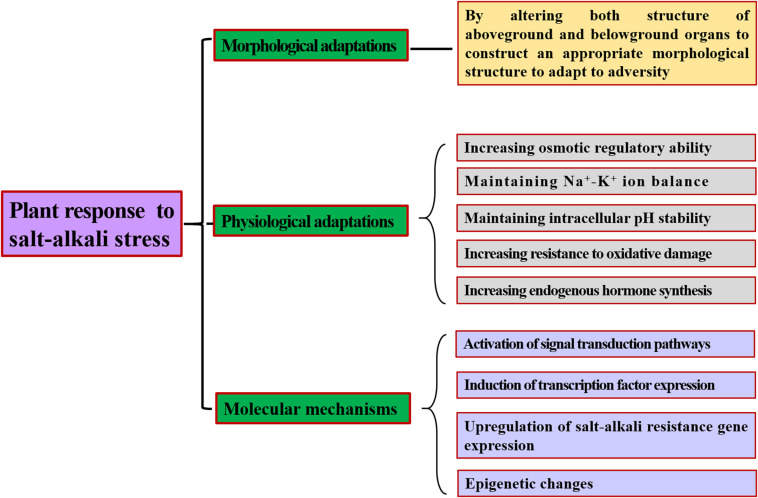
The response mechanism of plants to saline-alkali adversity.

Soil salinization has become a serious worldwide problem restricting the development of agroforestry. Research on the resistance mechanism of plants in response to saline-alkali stress is vital for selecting salt-tolerant varieties and utilizing saline land (Figure 2). Currently, studies are mainly focused primarily on salt stress and less on salt-alkali mixed stress. However, high salt and high pH often occur concurrently in nature, and their synergistic effect on plants is more harmful than the effect of either stress alone. Therefore, studying the resistance mechanisms of plants under mixed saline-alkali stress has more practical significance for cultivating new resistant varieties, screening new tolerance genes, and exploring new methods to improve plant tolerance to saline-alkali-stress conditions.

Saline-alkali-stress-tolerant crop breeding is a hopeful avenue for sustained agricultural development and the utilization of saline-alkali land. Many candidate genes have been cloned, and some genetically modified plants have been screened. However, the expression of these transgenes was not high, or their effect was not obvious, which may be related to the constitutive promoter used. In addition, the evaluation of tolerant transgenic plants has occurred mostly in laboratory or in greenhouse until now. It may not work well when these plants are exposed to the natural environment because of complex and variable field conditions and interactions with abiotic or biotic factors. Thus, there is still a long way to go. Nevertheless, with the development of modern biotechnology, especially molecular markers and gene-tagging methodologies, genome sequencing, microarray analysis and bioinformatic analysis, more tools and strategies can be applied to resolve the complex intriguing questions surrounding saline-alkali resistance.

## Author Contributions

SF and XL: conceptualization. XH: writing—original draft preparation. SF and XL: writing—review and editing. All authors contributed to the article and approved the submitted version.

## Conflict of Interest

The authors declare that the research was conducted in the absence of any commercial or financial relationships that could be construed as a potential conflict of interest.
